# Growth, Physiological, and Photosynthetic Responses of *Xanthoceras sorbifolium* Bunge Seedlings Under Various Degrees of Salinity

**DOI:** 10.3389/fpls.2021.730737

**Published:** 2021-09-27

**Authors:** Jian-Wei Zong, Zhi-Long Zhang, Pei-Lu Huang, Nai-Yu Chen, Ke-Xin Xue, Zhi-Yong Tian, Yu-Hua Yang

**Affiliations:** College of Art, Henan University of Animal Husbandry and Economy, Zhengzhou, China

**Keywords:** *Xanthoceras sorbifolium* Bunge, photosynthetic parameter, ion homeostasis, saline stress, antioxidant enzymes

## Abstract

*Xanthoceras sorbifolium* Bunge is priced for its medical and energetic values. The species also plays a key role in stabilizing ecologically fragile areas exposed to excess soil salinity. In this study, the effects of salinity on the growth, physiological, and photosynthetic parameters of *X. sorbifolium* Bunge were investigated. The *X. sorbifolium* seedlings were subjected to five salt treatments: 0 (control, CK), 70, 140, 210, and 280 mM of sodium chloride (NaCl) solutions. NaCl caused a decrease in plant height, specific leaf area, biomass, and root parameters. Leaf wilting and shedding and changes in root morphology, such as root length, root surface area, and root tips were observed. This study found that *X. sorbifolium* is tolerant to high salinity. Compared with the CK group, even if the concentration of NaCl was higher than 210 mM, the increase of the relative conductivity was also slow, while intercellular CO_2_ concentration had a similar trend. Moreover, NaCl stress caused an increase in the malondialdehyde (MDA), soluble proteins, and proline. Among the enzymes in the plant, the catalase (CAT) activity increases first and decreased with the increase in the intensity of NaCl stress, but the salt treatment had no significant effect on superoxide dismutase (SOD) activity. The peroxidase (POD) showed an increasing trend under salt stress. It was found that the photosynthesis of *X. sorbifolium* was notably impacted by saline stress. NaCl toxicity induced a noticeable influence on leaf net photosynthetic rate (Pn), stomatal conductance (Gs), intercellular CO_2_ concentration (Ci), transpiration rate (E), and water use efficiency (Wue). As salt concentration increased, the content of chlorophyll decreased. It can be found that a low concentration of NaCl induced the increase of photosynthetic capacity but a high-intensity exposure to stress resulted in the reduction of photosynthetic efficiency and SOD activity, which had a positive correlation. In summary, salt-induced ionic stress primarily controlled root morphology, osmotic adjustment, and enzyme activities of salt-treated *X. sorbifolium* leaves, whereas the low salt load could, in fact, promote the growth of roots.

## Introduction

High salinity is a major problem in arid and semiarid tropics (Allard et al., 1998; Mahajan and Tuteja, 2005). Salt-affected soils are distributed worldwide, and every continent is faced with this challenge (Brady and Weil, 2016). Approximately, 20% of the total cultivated area of the world and nearly 50% of irrigated lands have been degraded by salinity (Yeo, 1999; Tuteja, 2007). With the increase of the population pressure, these problems also occur in China; therefore, the improvement and utilization of saline-alkali land have become a focus for the economic development of agriculture production in China. Therefore, it is increasingly necessary to cultivate salt-tolerant plants using cost-effective strategies that can be applied at a large scale.

*Xanthoceras sorbifolium* Bunge, also known as a yellow horn, is a kind of deciduous perennial shrubs or small trees of the Sapindaceae family and the monotypic genus *Xanthoceras* (Wang et al., 2018). It is one of the unique ecological oil species in northern China and is listed as one of the important tree species for biomass energy production. Having outstanding energy values, medicinal values, and greening values, *X. sorbifolium* Bunge can be pressed for oil and is edible, medicinal, and ornamental. The species is considered an important bioenergy tree for biomass energy production due to its abundant content of seed kernel oil (55–65%), which is rich in unsaturated fatty acids (85–93%) (Wang et al., 2015). It has vigorous vitality and can grow healthily under various environments (Zhou and Cai, 2018). The plant could develop and complete its life cycle normally under the saline and alkaline soil (above 70 mmol·L^−1^ monovalent salt) in northern China, where the temperature can be below −40°C, making it suitable for wide use in a wretched environment. At present, many developing regions are facing the problem of “larger population and less land.” As a consequence, the development of biomass energy is restricted by the reduction of cultivated land resources. If *X. sorbifolium* Bunge can be widely planted in saline soils, it would provide valuable solutions for addressing the challenges of energy and ecology security.

Numerous studies have reported on the effect of salt stress on leaves and roots, and their results revealed that salt stress exerted distinct effects on growth, photosynthesis, membrane permeability, osmotic adjustment substance, enzyme activity, and so on (Sorkheh et al., 2012; Kargbo et al., 2019; Yang et al., 2020). In addition, researchers have provided important insights into salt and saline-alkali stress tolerance of yellow horn (*X. sorbifolium*) on physiological and transcriptomic levels (Wang et al., 2020). In the study, Zhang X. Y. et al. (2013) compared the effects of five concentrations of sodium chloride (NaCl) on *X. sorbifolium*, and it was found that the plant could alleviate the osmotic stress induced by NaCl solution, proving the salt tolerance of the species. Another study (Chen, 2017) also reported that the different damage of lipid membranes of *X. sorbifolium* may be explained by different saline-alkaline treatment and duration time, as the plants under 200 mM NaCl had higher electrolyte leakage than the control (CK) group. However, the property of salinity tolerance is not a simple attribute, but it is an outcome of various features that depend on different physiological interactions, which are difficult to determine. In this study, the growth conditions, root morphology, enzyme activities, and photosynthesis were focused on, according to the abovementioned studies. There will be a comprehensive investigation on the salt tolerance of this plant in the long term.

Many strategies have been used to improve the salt tolerance of plants. Attempts have been made in the past to understand the effects of salinity by observing plant growth regulators. The root system is the key vegetative organ connecting a plant and its underground environment. Not only can the root system anchors plants and absorbs and transports water and nutrients (Miao et al., 2020) but also it plays an important role in sensing salt stress. Generally, salt-tolerating plants are characterized by low transportation rates of sodium ion (Na^+^) to the shoot, keeping aboveground parts receiving Na^+^ as free as possible (Munns et al., 2000). The relationship between shoot and root of plants was observed in this study. In addition, it has been reported that the osmotic and water-deficit-inducing effects of salinity lead to a reduction of leaf photosynthesis and growth, which can be overcome by osmolyte accumulation such as proline and soluble proteins (Munns et al., 2012). Moreover, peroxidase (POD), catalase (CAT), and superoxide dismutase (SOD) are enzymes responsible for the scavenging of reactive oxygen species (ROS) (Apel and Hirt, 2004). It is proved that the physiological parameters can reflect the overall condition of plants under salt stress, so relevant explorations were also arranged in the experiment. Photosynthesis is one of the primary processes to be affected by salt stress (Chaves et al., 2009), and relative symptoms can be witnessed easily on the leaves.

The salt-induced reduction of photosynthesis has been reported to be associated with several factors (Mishra and Das, 2004), which can be roughly classified into stomatal factors and non-stomatal factors. It was found that the photosynthesis decline of lettuce under salt stress is mainly caused by non-stomatal factors (Xie et al., 2018), and this regulation was also tested in the experiment.

Taking into account prior studies, the major objective of this study was as follows:

(1) To observe the physiological, photosynthetic, root, and overall responses of *X. sorbifolium* Bunge plant to salt stress and the salt-tolerance mechanisms and to understand whether the changes of these parameters could be related to their degree of salt tolerance.
(2) To select the most suitable salt concentration for the growth of the plant and to discuss how this plant can be used to improve saline land.

## Materials and Methods

### Site Description, Plant Materials, and Growth Conditions

The experiment was conducted in a seedling nursery land in Zhengzhou (113″80′ E, 34″80′ N), northern China, from December 2018 to August 2019. The climate of Zhengzhou is semi-humid with an average of 220 frost-free days. Approximately, 80% of the annual precipitation of 640.9 mm falls from May to September, and the mean annual temperature is 14.4°C. The seeds were bought from YangLing Jinshan Agricultural Science and Technology Co. Ltd. and then were buried in the sand at low temperature. The seeds are called “*Xanthoceras sorbifolium* Bunge 1,” and the accession number is 6853202 (http://www.jinshan01.com/wgg/237713.html).

*Xanthoceras sorbifolium* Bunge seeds were sowed in December 2018. In March 2019, the healthy and homogenous seedlings were dug up and transferred to 2-kg plastic flowerpots (inner diameter: 28 cm; height: 20 cm; comprising a bottom pierced with holes; one seedling per pot) containing 1.865 kg of the substrate (volume ratio of peat to perlite is 6:1). Then, they were placed in the rain shelter, with unified management and regular weeding and watering.

### NaCl Treatment

After a period of cultivation, 45 seedlings with optimal growth conditions and the same growth potential and plant height of about 30 cm were randomly selected for saline exposure in July 2019. The seedlings were divided into five groups according to the salt concentration treatments, with three replications assigned to each treatment. They were exposed to five NaCl stress treatments as follows: 0 (CK), 70, 140, 210, and 280 mM. The stress time was calculated from the last day of adding the salt solution, which sustained a total of 50 days. The CK group was watered with distilled water, and the treatment groups were added with the corresponding treatment solution (irrigated three times, once in every 3 days, and 300 ml per pot for each time). To help avoid solution loss, the pot tray was used so that solutions permeated through the pots can be poured back. The duration of the treatments was 50 days, and all the plants survived till the end of the treatments.

### Plant Growth Parameters and Ion Characters

Relative height rate was calculated from the increase in plant height measured at the beginning and end of the salinity stress, where *H* is the plant height and *T*1–*T*0 is the time interval or relief periods (Tattini et al., 2002).


(1) $$\begin{array}{r} Relative\;height\;rate\left(cm/day\right)=\frac{H1-H0}{T1-T0} \end{array}$$

To determine the biomass production, all the potted plants were completely pulled out of the pots, and the shoot and root tissues were cleaned with running water. Each plant sample was divided into three parts, namely, leaf blade, stem (including the leaf sheath), and root. The specific leaf area was calculated by dividing the leaf area by the corresponding fresh leaf weight. Three plants from each treatment group were randomly taken to measure the root biomass and shoot biomass. Dry weight (DW) was recorded after fully drying at 80°C for 48 h. The average values of these parameters were calculated, and the root–shoot ratio was calculated using the following formula:


(2) $$\begin{array}{r} Root-shoot\;ratio\left(\%\right)=\frac{Ground\;dry\;weight}{Underground\;dry\;weight} \end{array}$$

Concentrations of Na^+^ and K^+^ were determined following the method of Chen et al. (2017) and Chen J. et al. (2017). In brief, oven-dried roots were finely ground in a sample mill (model Label, Miser LM-Plus; Osaka Chemical Co., Ltd., Japan), and the fine power was acid-digested with 8 ml of HNO_3_ + 2 ml of HClO_4_. The Na^+^ and K^+^ concentrations of the extracts were then measured using a flame photometer (ANA 135; Tokyo Photoelectric; Tokyo, Japan).

### Root Morphology Analysis and the Activity of the Root System

Three plants of each treatment were used to determine the values of the root parameters. A piece of absorbent paper was used to wipe the surface of the root, and its weight was recorded as the fresh weight (FW). Then, each root system was scanned by using the EPSON TWAIN PRO root scanner (32 bit, Canada Regent Instrument Inc., Canada) and analyzed its length, surface area, system volume and tips, diameter, and projected area (WinRHIZO 2005, a root analysis software).

Root activity was determined using the 2,3,5-triphenyltetrazolium chloride (TTC) method (Berry et al., 2010). In brief, the root sample (0.1 g) was taken as the CK, to which 2 ml of sulfuric acid (H_2_SO_4_, 1 M) was added. Other samples (0.1 g) were mixed with 5 ml of 0.4% (w/v) TTC and 5 ml of potassium phosphate buffer (66.7 mM, pH 7.0), and the mixed liquids and CK group were incubated at 37°C for 4 h. Later, sulfuric acid was added to the mixed liquids to stop the reaction. Fifteen minutes later, the root samples were dried and placed in clean tubes with 10 ml of 95% (w/v) ethanol for 24 h until the roots turned white. The absorbance of extract solution was measured at 485 nm, and the root activity was calculated according to the standard curve which was prepared in advance.

### Lipid Peroxidation and Membrane Permeability

For the measurement of electrolyte leakage, fresh leaves were punched and soaked in sterile water at 4°C for 2 h. The first conductivity value was measured using a conductivity meter DDS-307 (Leici Corporation, China) and named *L*1. The homogenate was boiled at 100°C in a water bath for 20 min and cooled down to room temperature. Then, the second conductivity value was recorded and named *L*2 (Ahmad et al., 2018). The relative electrical conductivity (REC), representing the degree of electrolyte leakage, was calculated by using the following formula:


(3) $$\begin{array}{r} Relative\;Electrical\;Conductivity\left(\%\right)=\frac{L1}{L2}\times 100\% \end{array}$$

The method suggested by Garriga et al. (2015), with slight modifications, was used to estimate malondialdehyde (MDA). In brief, 0.5 g of a fresh sample of the leaf was homogenized in 10 ml of 0.1% trichloroacetic acid solution (TCA). The extract obtained was then centrifuged at 14,000 × *g* for 7 min. Of note, 1.5 ml was taken from the supernatant, thoroughly mixed with 0.5% of thiobarbituric acid and 6 ml of 20% TCA, heated up to 95°C for 30 min, and then cooled on an ice bath. The mixture was then centrifuged at 10,000 × *g* for 10 min, and the absorbance of the supernatant was recorded at 532 and 660 nm.

### Antioxidant System and Soluble Accumulation

The leaf samples were ground with a mortar and pestle under chilled conditions in a homogenization buffer. The homogenate was centrifuged at 10,000 × *g* for 20 min at 4°C, and the supernatants were used for the enzymatic assays. POD activity in the leaves was estimated with a method described by Thomas et al. (1982) using guaiacol as the substrate. In brief, the guaiacol POD activity was measured with guaiacol as the substrate in a total volume of 3 ml. The reaction mixture consisted of 50 mM potassium phosphate buffer (pH 6.1), 1% guaiacol, 0.4% H_2_O_2_, and enzyme extract. The increase in the absorbance due to the oxidation of guaiacol was measured at 470 nm. One unit of POD activity was defined as the 470 nm value reduced by 0.01 in 1 min.

The activity of CAT was determined by following the method of Luck (1974). The activity of CAT was calculated using the extinction coefficient of 36 × 103 mM·cm^−1^ and expressed as enzyme unit (EU) g^−1^ protein. The SOD activity was measured using the *o*-methoxy-phenol method suggested by Van Rossum et al. (1997). SOD activity is inversely proportional to the Nitro Blue Tetrazolium Chloride monohydrate (NBT) reduction. SOD unit is the amount of protein that restricts 50% of the photoreduction of NBT. SOD activity was expressed as EU mg^−1^ protein.

Soluble protein content was determined following the method of Lowry et al. (1951), using bovine serum albumin as the standard. In brief, 0.5 g of a fresh sample was taken, and 2 ml of distilled water was added for grinding. After the homogenate was ground, the mortar was washed with 6 ml of distilled water. The washing liquid was collected in the same centrifuge tube and centrifuged at 4,000 × *g* for 10 min. The resulting supernatant was transferred into a 10-ml volumetric flask, which was then filled with water and then shaken evenly. Later, 0.1 ml of the sample extract was aspirated into a stopper tube. A solution of 5 ml of Coomassie bright blue G-250 was added and mixed thoroughly. After being placed for 2 min, the absorbance was measured at 595 nm by using a multiple wavelength spectrophotometer (UV-2100, UNIC, USA).

The proline content in matured leaves was measured by using the rapid colorimetric method as suggested by Bates et al. (1973). Proline was extracted from 0.5 g of leaf samples by grinding in 10 ml of 3% sulphosalicylic acid, and the mixture was then centrifuged at 10,000 × *g* for 10 min. Then, 2 ml of this supernatant and 2 ml of freshly prepared acid-ninhydrin solution were added into each test tube. These tubes were incubated in a water bath at 90°C for 30 min. The reaction was terminated in an ice bath. The proline concentration in the sample was determined from a standard curve using analytical grade proline (SRL, Mumbai, India) and calculated on the FW basis.

### Photosynthetic Parameters and Photosynthetic Pigments

Net photosynthetic rate (Pn), stomatal conductance (Gs), transpiration rate (E), and intercellular CO_2_ concentration (Ci) of the leaves were determined at 08:30–10:30 on fully expanded leaves, using photosynthesis-apparatus Li-6400 (LI-COR Inc., Lincoln, NE, USA) that maintains photosynthetic photon flux density (PPFD) at 1,200 μmol·m^−2^·s^−1^ and CO_2_ concentration at 400 μmol·mol^−1^ (Zhang et al., 2015; Kiyomizu et al., 2018).

The chlorophyll content in the leaves was measured using previously established methods (Li et al., 2015). The third or fourth leaves below the plant buds were taken for pigment sampling at the end of the experiment. The Chla, Chlb, and Car contents were determined in 80% acetone extract using a UV-2100 spectrophotometer (UNIC, USA).

### Statistical Analyses

Three technical repeats were performed for each assay, and the results were reported as mean and SE. The data were processed using SPSS 25.0 software (SPSS, Chicago, USA) for statistical and principal component analyses. One-way ANOVA was performed to identify statistically significant differences among treatments, followed by Duncan's multiple range test at *P* < 0.05. Pearson's correlations were drawn between physiological and photosynthetic parameters. These data were also included in principal component analyses, and the charts were made using Origin 8.0 software (ORIGIN, Massachusetts, USA).

## Results

### Growth Characteristics

Salinity significantly (*P* < 0.05) adversely affected the growth of *X. sorbifolium* Bunge (Figure 6), as indicated by changes in parameters such as the biomass production, root–shoot ratio, shoot DW, root DW, and relative height rate. The response of plant growth to salinity stress varies with NaCl concentrations (Table 1). Compared with the control values, the biomass production of the plant was obviously inhibited under the treatment of the high concentration of salt. Additionally, with the increase of salt concentration, shoot DW, root DW, and the increasing rate of plant height all showed a trend of reduction, while the specific leaf area did not show any significant change (*P* > 0.05). Under the treatment of 280 mM salt, shoot DW, root DW, relative height rate, and total DW was 11.9, 23.7, 56.4, and 16.4%, respectively, lower than those of the control samples. Under the treatment of 210 mM salt, the highest reduction was observed in biomass (15.7%), followed by shoot DW (11.4%) compared with controls. Furthermore, the root–shoot ratio of *X. sorbifolium* increased as salt concentration increased, and the increase was statistically significant (*P* < 0.05), suggesting that NaCl stress inhibits the stem and leaves more than the roots.

**Table 1:** Effect on some growth parameters of *Xanthoceras sorbifolium* seedlings under sodium chloride (NaCl) stress.

**Treatment**	**Relative height rate**	**Shoot dry weight**	**Root dry weight**	**Biomass production**	**Root to shoot ratio (%)**	**Specific leaf area**
**(mM)**	**(cm·d^**−1**^)**	**(g·plant^**−1**^)**	**(g·plant^**−1**^)**	**(g·plant^**−1**^)**		**(cm^2^·g^**−1**^DW)**
0	0.101 ± 0.004a	14.4 ± 0.39a	23.35 ± 0.41a	37.75 ± 0.79a	1.29 ± 0.09b	113.32 ± 3.57a
70	0.081 ± 0.002b	12.04 ± 0.18b	21.53 ± 0.15b	33.58 ± 0.33b	1.40 ± 0.01b	110.46 ± 2.06a
140	0.063 ± 0.002c	11.82 ± 0.22b	21.11 ± 0.28bc	32.93 ± 0.50bc	1.86 ± 0.13ab	108.85 ± 1.82a
210	0.044 ± 0.004d	10.90 ± 0.32c	20.68 ± 0.09c	31.83 ± 0.22c	1.82 ± 0.24ab	110.69 ± 1.45a
280	0.019 ± 0.003e	10.99 ± 0.04c	20.57 ± 0.07c	31.56 ± 0.06c	1.91 ± 0.21a	108.98 ± 3.22a

*The values presented the means ± SE. Different letters indicate significant differences between irradiance treatments (P < 0.05)*.

### Root Morphology Parameters and Ion Toxicity

A similar manner of results was investigated for the root parameters. With NaCl supplement, the highest values were all observed in the 70 mM group. Furthermore, the minimum reduction in the root length was recorded in 140 mM (7.9%) followed by 210 mM (29.6%), and the maximum reduction was observed in 280 mM (37.6%) as compared with their respective controls (Table 2).

**Table 2:** Effect on some root parameters of *X. sorbifolium* seedlings under NaCl stress.

**Treatment**	**Root diameter**	**Root length**	**Root surface area**	**Root projected area**	**Root volume**	**Root tips**	**Root activity**
**(mM)**	**(mm)**	**(cm·plant^**−1**^)**	**(cm^2^·plant^**−1**^)**	**(cm^2^·plant^**−1**^)**	**(cm^3^·plant^**−1**^)**		**(μg·g^**−1**^ FW·h^**−1**^)**
0	1.08 ± 0.04a	827.86 ± 108.84b	229.07 ± 41.91ab	72.92 ± 13.34b	19.49 ± 3.81ab	4168.00 ± 604.00b	0.14 ± 0.10a
70	0.95 ± 0.00a	1428.40 ± 60.52a	346.34 ± 15.28a	110.24 ± 4.86a	25.63 ± 0.45a	8000.00 ± 34.00d	0.06 ± 0.02a
140	0.81 ± 0.11a	762.12 ± 73.98b	160.48 ± 8.41b	51.08 ± 2.68bc	11.37 ± 0.94b	4714.00 ± 466.00b	0.05 ± 0.00a
210	0.78 ± 0.08a	583.00 ± 72.80b	120.66 ± 24.99b	38.41 ± 7.95c	8.34 ± 2.58b	3414.00 ± 437.00bd	0.06 ± 0.03a
280	0.84 ± 0.20a	516.04 ± 202.98b	120.14 ± 67.02b	17.89 ± 0.98c	9.79 ± 5.89b	1280.50 ± 426.50c	0.04 ± 0.01a

*The values presented the means ± SE. Different letters indicate significant differences between irradiance treatments (P < 0.05)*.

High salinity levels induced significant (*P* < 0.05) increases in the Na^+^ content over the CK group. In addition, the K^+^ content in the root showed a remarkable increase under the low salinity level (70 and 140 mM), with the maximum observed at 140 mM. After salt treatment, the K^+^/Na^+^ content of the plant roots decreased significantly, by 68.4% (Table 3), as compared with the CK group.

**Table 3:** Effect on root K^+^, Na^+^, and K^+^/Na^+^ ratio of *X. sorbifolium* seedlings under NaCl stress.

**NaCl (mM)**	**K^**+**^ (mg·g^**−1**^)**	**Na^**+**^ (mg·g^**−1**^)**	**K^**+**^/Na^**+**^**
0	5.23 ± 0.083c	2.88 ± 0.063e	2.25 ± 0.031a
70	6.48 ± 0.07b	3.87 ± 0.059d	2.18 ± 0.055a
140	8.44 ± 0.089a	3.43 ± 0.178c	1.53 ± 0.093b
210	4.89 ± 0.028d	4.6 ± 0.14b	1.06 ± 0.03c
280	4.16 ± 0.044e	5.87 ± 0.078a	0.71 ± 0.017d

*The values presented the means ± SE. Different letters indicate significant differences between irradiance treatments (P < 0.05)*.

### Cell Damage and Osmotic Adjustment

**Figure 1:**
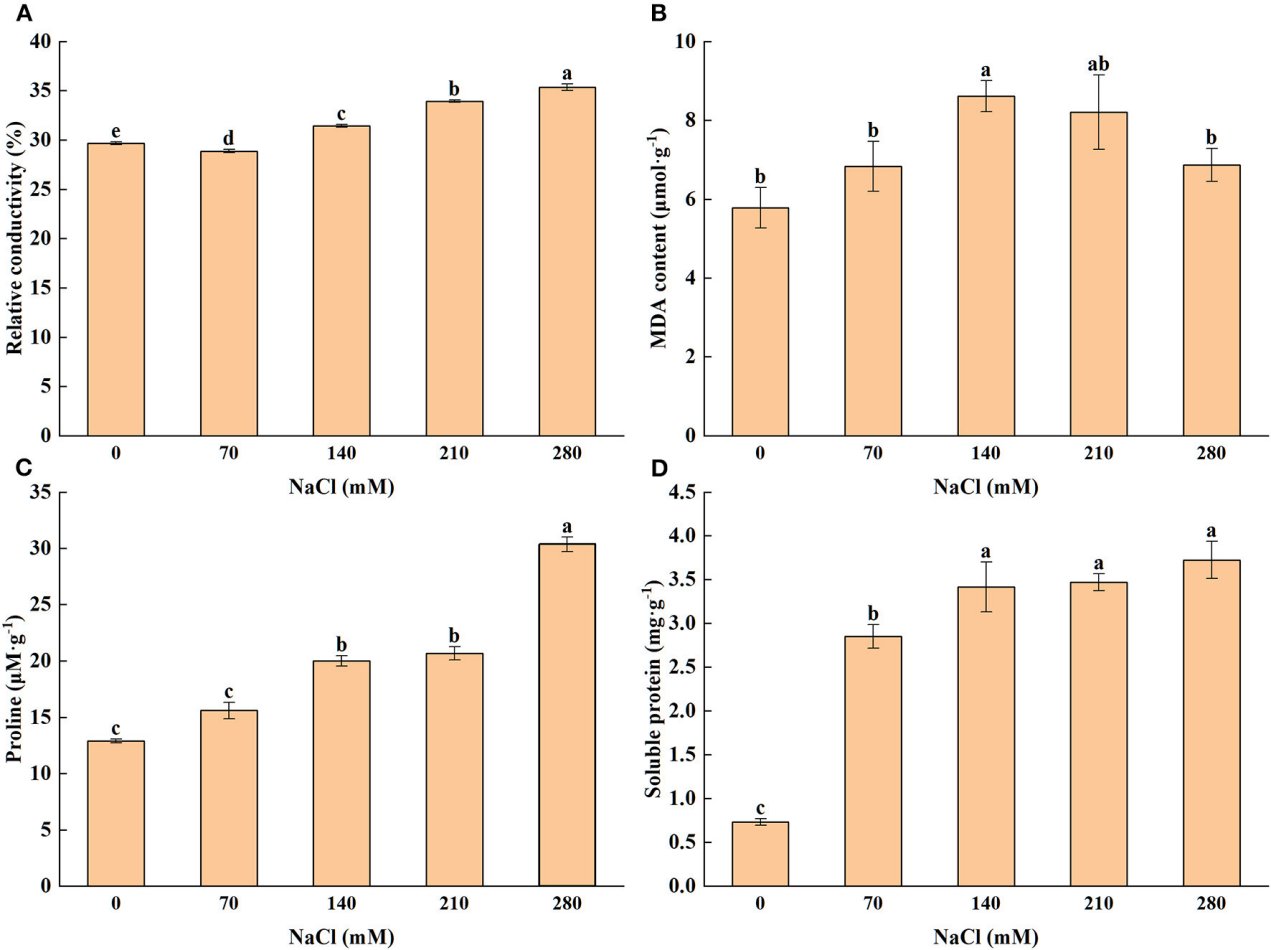
Levels of relative electrolyte conductivity **(A)**, malondialdehyde content **(B)**, proline **(C)**, soluble protein **(D)** subjected to different levels of salt stress [0, 70, 140, 210, and 280 mM sodium chloride (NaCl)]. Means (*n* = 6 per treatment ± SE) with at least one same letter are not significantly different at *P* < 0.05.

An increase in the concentration of salt caused a gradual increase in the relative conductivity of the leaf. The result proved that even under a higher concentration of salt, the value of relative conductivity was also stable (Figure 1A). Similarly, under the controlled condition, the significant influence was noticed for the MDA content under different salt concentrations (Figure 1B), with 140 and 210 mM of NaCl exhibiting the largest MDA (8.61 and 8.21 μmol·g^−1^, respectively). Seedlings were grown under the CK condition induced the lowest MDA content.

Proline is one of the most common osmotic adjustment substances, whose content depends on the concentration of NaCl in this experiment. Results (Figure 1C) indicated that leaf proline concentration enhanced notably with increasing salt stress. The content of soluble protein, an osmotic adaptive parameter, was also assayed, and the results showed that the highest (3.724 mg·g^−1^) and lowest (0.732 mg·g^−1^) values occurred at 280 and 0 mM of NaCl, respectively (Figure 1D).

### Antioxidant Enzyme Activities

**Figure 2:**
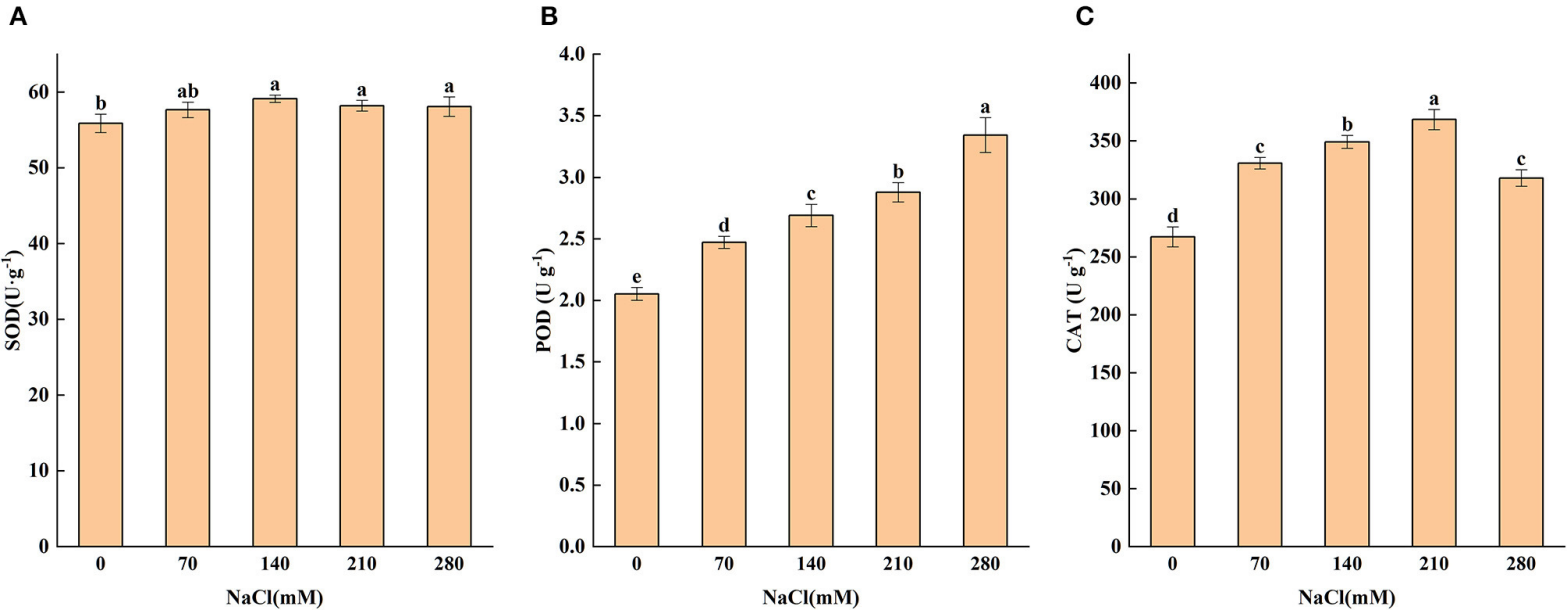
Levels of superoxide dismutase **(A)**, peroxidase **(B)**, and catalase **(C)** subjected to different levels of salt stress (0, 70, 140, 210, and 280 mM NaCl). Means (*n* = 6 per treatment ± SE) with at least one same letter are not significantly different at *P* < 0.05.

Antioxidants play an essential role in responding to salt stress by inhibiting oxidation. Salt-stress substantially enhanced the POD activity of seedling leaves, and the largest value occurred at 280 mM. When the salt concentration varied from 70 to 210 mM, the POD activity significantly (*P* < 0.05) increased by 20.4, 31.2, 40.0, and 62.9%, (Figure 2B) respectively, as compared with control, indicating that salt stress may have induced the plant to produce a certain amount of POD to remove excessive peroxide, consequently improving the salt tolerance of the plant.

Catalase activity under NaCl stress was higher than that in the CK group across all treatments (Figure 2C). It can be seen that 70 and 140 mM of NaCl treatments enhanced CAT activity by 23.8 and 30.7%, respectively, in comparison with the CK group. From 70 to 210 mM of NaCl treatment, a continuous increase was observed in CAT activity; but when NaCl treatment further increased to 280 mM, CAT activity dropped to a level below that of 70 mM treatment but still significantly above the CK group. It can be seen that SOD did not change significantly under high NaCl concentration (Figure 2A), but the POD and CAT did. It could be concluded that higher activities of POD and CAT in salt-stressed leaves may protect the plant tissues from membrane oxidative damage under salt stress, thus alleviating salt toxicity and improving the growth of the plant.

### Photosynthetic Parameters and Photosynthetic Pigments

**Figure 3:**
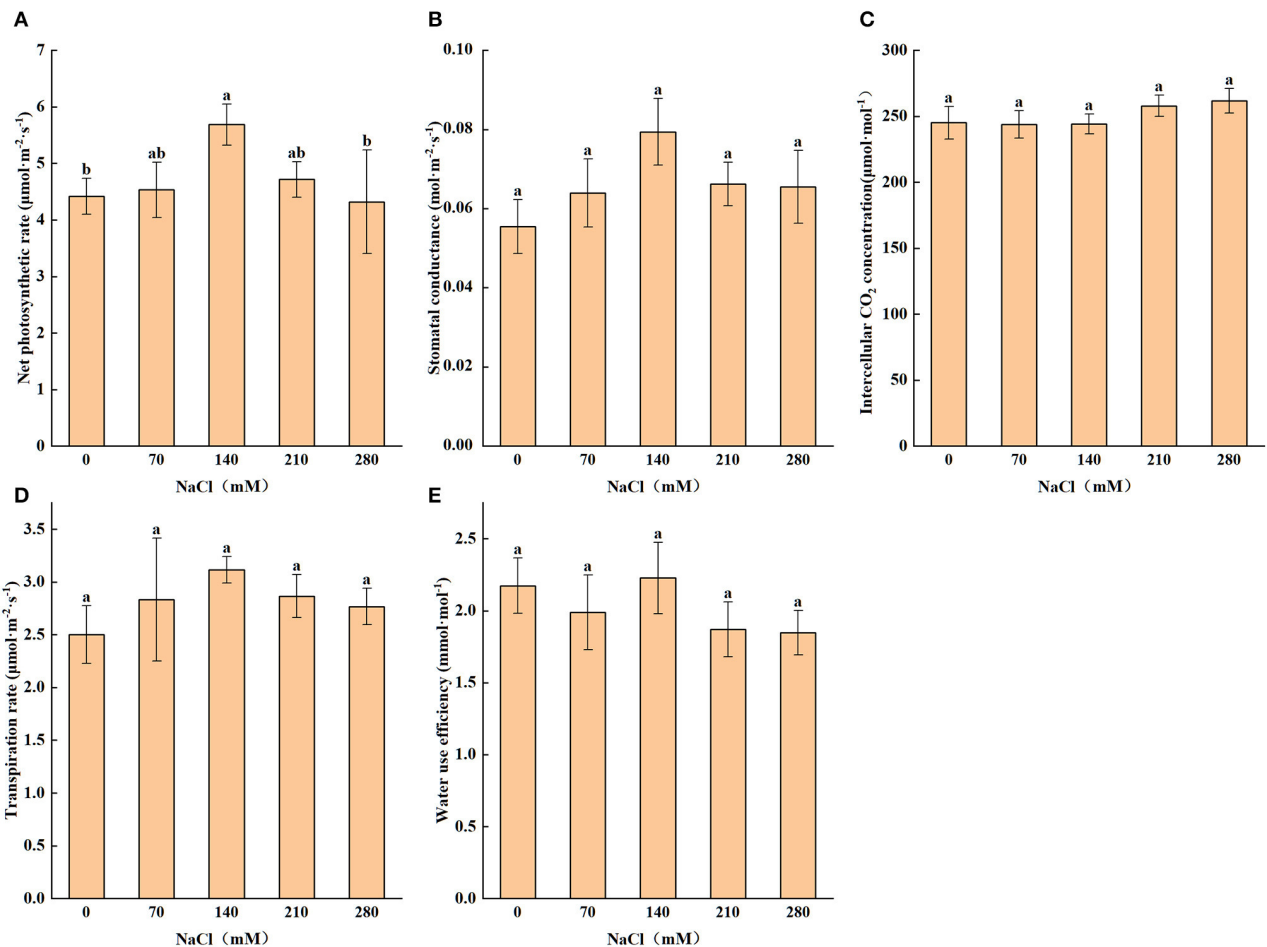
Levels of net photosynthetic rate **(A)**, stomatal conductance **(B)**, transpiration rate **(C)**, intercellular CO_2_ concentration **(D)**, water use efficiency **(E)** subjected to different levels of salt stress (0, 70, 140, 210, and 280 mM NaCl). Statistics as shown in Figure 1.

Salt stress has a significant impact on the photosynthesis of *X. sorbifolium*. As shown in Figure 3, Pn, Gs, and transpiration rate (E) were highest at 140 mM NaCl and then notably (*P* < 0.05) decreased when the concentration was greater than it. The pattern of change is similar for these photosynthetic parameters, and the maximum values of them were 28.72, 43.32, and 24.38%, respectively, higher, as compared with the control values. The change of Ci is not significant across the concentrations (*P* > 0.05). Moreover, water use efficiency (Wue) also reached the highest point under the 140 mM NaCl treatment.

With the increase of salt concentration, Chla and Chlb significantly decreased by 32.2 and 49.2%, respectively, compared with the CK (Table 4). At 70 mM, the content of car and total chlorophyll increased slightly, and Chla/b increased significantly, which may be caused by the promoting effect of low concentration of salt on the plant.

**Table 4:** Effect on photosynthetic pigment of *X. sorbifolium* seedlings under NaCl stress.

**Treatment**	**Chla**	**Chlb**	**Car**	**Chal a/b**	**Total pigment**
**(mM)**	**(mg·g^**−1**^)**	**(mg·g^**−1**^)**	**(mg·g^**−1**^)**	**(mg·g^**−1**^)**	**(mg·g^**−1**^)**
0	2.76 ± 0.06a	1.24 ± 0.04a	10.24 ± 0.07c	2.22 ± 0.04c	14.24 ± 0.06ab
70	2.67 ± 0.04a	0.93 ± 0.14b	11.06 ± 0.02a	2.94 ± 0.01b	14.67 ± 0.02a
140	2.42 ± 0.03b	0.8 ± 0.16b	10.76 ± 0.16b	3.01 ± 0.07b	13.98 ± 0.14b
210	2.38 ± 0.02b	0.65 ± 0.41c	8.83 ± 0.14d	3.64 ± 0.02a	1·1.86 ± 0.06c
280	1.87 ± 0.09c	0.61 ± 0.31c	8.27 ± 0.21e	3.07 ± 0.1b	10.75 ± 0.21d

*The values presented the means ± SE. Different letters indicate significant differences between irradiance treatments (P < 0.05)*.

### Principal Component Analysis and Pearson's Correlation Analysis

**Figure 4:**
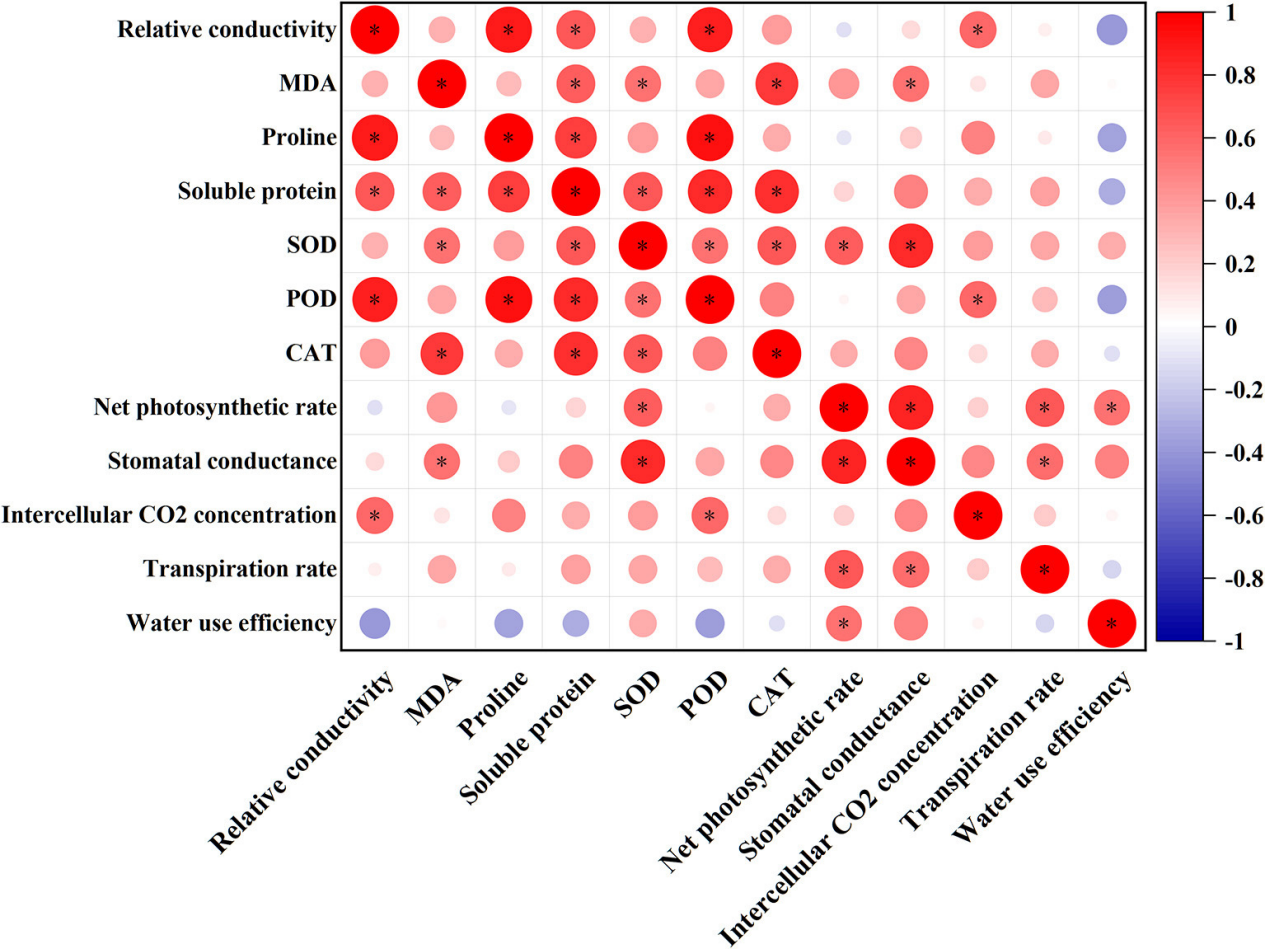
Correlation analysis of physiological, photosynthetic, and antioxidant enzyme activities of *Xanthoceras sorbifolium* Bunge seedlings in response to salt stress. *Significant at the 5% levels and color depth represents the correlation coefficient.

Figure 4 shows the correlation coefficients between physiological and photosynthetic indices by Pearson's correlation analysis. In this study, SOD was positively correlated with CAT and Pn, while POD was negatively correlated with REC, proline, and soluble proteins. In addition, REC, Ci, MDA, and Gs also showed a significant positive correlation.

**Figure 5:**
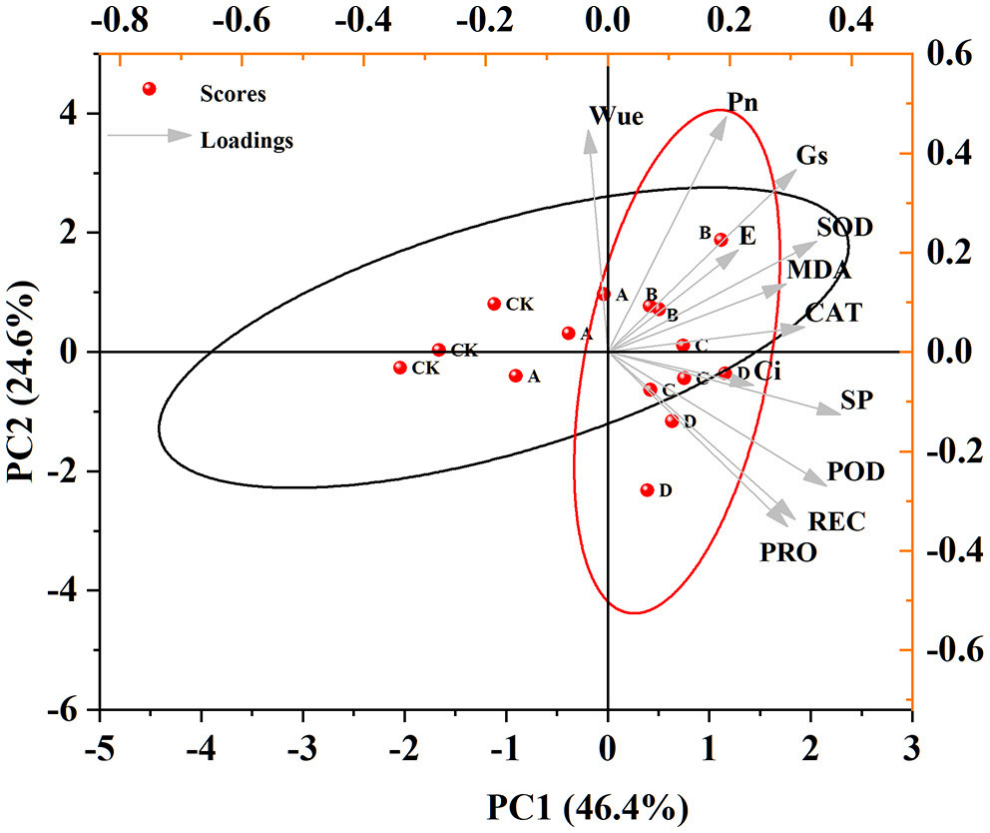
Principal component analysis for physiological, photosynthetic, and antioxidant enzyme activities under salt stress. “SP” represents soluble protein. “PRO” represents proline. Mean values for each species were used for the analysis (*n* = 3). “A” represents the 70 mM NaCl treatment group, “B” represents the 140 mM NaCl treatment group, “C” represents the 210 mM NaCl treatment group, and “D” represents the 280 mM NaCl treatment group.

Principal component analysis (PCA) was conducted that picked out two principal components from 12 indices where the first and second components were 46.4 and 24.6% of the total variance (71.0%) as biplots (Figure 5, Supplementary Tables 1, 2). Salt-treated groups at the level of 0 and 70 mM were mostly located at the left side of the plot and had a strong negative correlation with the first component. The salt-treated group at the level of 140 mM was located on the upper right side of the plot while those treated with 210 and 280 mM of NaCl were mostly located on the lower right side of the plot. The salt-stressed group that was located at the first ellipse (140 mM) had a highly negative correlation with the second component. The first component had positive correlations with other parameters except for Wue. The second component had strong positive correlations with Wue, Pn, Gs, SOD, MDA, E, and CAT and had negative correlations with Ci, soluble proteins, POD, proline, and REC. The groups that had a positive correlation with the first and second components were considered to be more tolerant to salt stress. In this study, the 140 mM-treated group was the unique one that had a positive correlation with both components. In other words, it was found that the plant species grew better and expressed various best-performing indicators under the 140 mM salt treatment.

**Figure 6:**
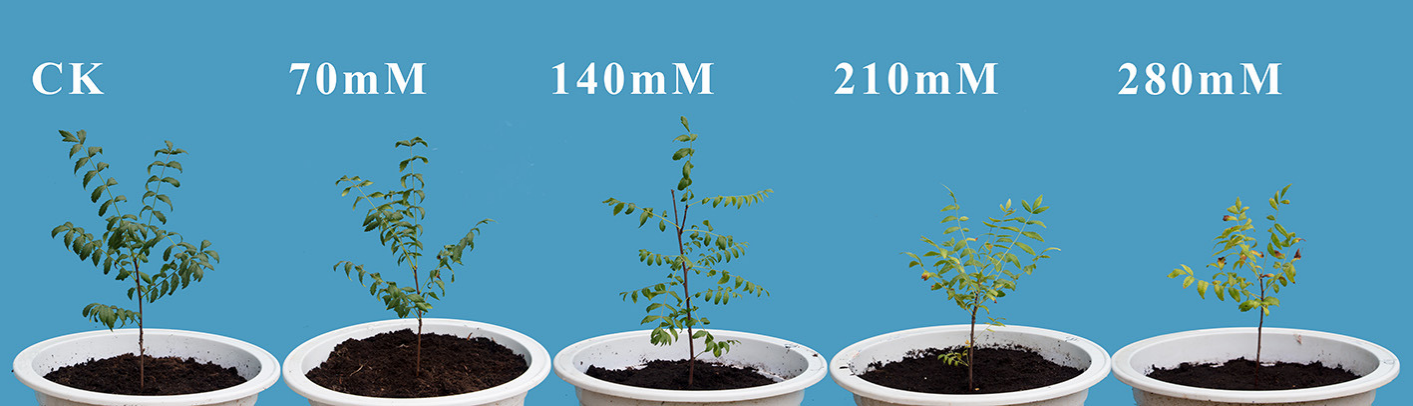
Leaves of *X. sorbifolium* Bunge seedlings grown under various NaCl concentrations (0, 70, 140, 210, and 280 mM).

## Discussion

It is generally recognized that salt stress plays an important role in various aspects of physiology and biochemistry (Gottschalk, 1994). Disturbance of ionic balance resulting from Na^+^ overload inside of cells causes osmotic stress and growth restriction (Pessarakli, 1999). Plants can deal with the saline condition either by tolerating it or by avoiding it (Xie et al., 2008). In this study, the growth parameters, morphology, photosynthetic capacity, and physiology characteristics were examined to scan the salt tolerance of *X. sorbifolium*.

The response of plants to excessive salt is usually characterized by a decline in plant growth (Hauser and Horie, 2010). The interference of various physiological and biochemical processes at the cell, tissue, or whole plant levels, such as photosynthesis, nutrient uptake and accumulation of compatible solutes, and enzyme activity, has a massive impact on the growth and survival of plants (Fu, 2004; Sofo et al., 2005; Munns and Tester, 2008). The results of this study indicated that the DW and relative height rate were significantly hindered under salinity stress, while the specific leaf area was not. It was speculated that the NaCl stress mainly caused leaf wilting rather than a decrease in leaf area since the leaf area of *X. sorbifolium* is small as compared with other trees.

Although the growth reduction was noticed in both the shoot weight and root length at the high salinity level, it can be seen that the low level of salt (70 mM) can promote the increase of the root length and root tip, while the root surface area and root volume have a similar trend. A recent study also showed that under conditions of brackish water drip irrigation, the leaching of salt from the root zone was improved and a wider and deeper root system developed (Berezniak et al., 2017). This suggested that a low concentration of NaCl is beneficial to the growth of root, or more exactly, fibrous roots, which is more efficient to improve absorption activity. Furthermore, the Na^+^, K^+^ in the root, and the origin of ionic stress can reflect the salt tolerance of the plant. It was found that Na^+^ increased rapidly, while K^+^ has a significant reduction with the increase of NaCl concentration. In another study, it is the high concentration of Na^+^ in the soil environment that inhibits the absorption of K^+^ and promoted the outflow of K^+^ (Daneshmand et al., 2010). As the most direct part to cope with the salinity, roots could regulate and balance their functions under NaCl stress. This study found that the root surface area of the root system reduced, likely to limit the absorption of Na^+^ that could lead to the occurrence of ion poisoning due to the salt stress. The ability of the root system to absorb water and transport nutrients was limited, thus affecting plant growth (Patel and Pandey, 2008).

Meanwhile, the contents of REC, MDA, proline, soluble proteins, and antioxidant enzyme activities increased differently under salt stress. MDA, resulting from the lipid peroxidation of polyunsaturated fatty acids, is an indicator of free radical production and consequent tissue damage (Mahajan and Tuteja, 2005). Under stress conditions, the plant cell membrane is damaged by salt stress, and a great quantity of MDA is produced. In addition, ROS formation occurs during photosynthetic light reactions, and the process of scavenging ROS could arise from oxidative damage (Wang et al., 2004). In this study, the MDA content was notably larger under salt stress than the CK group that was not treated with salt and reached the lowest value under 280 mM, indicating greater damage by ROS. At this time, the protective enzyme systems (i.e., SOD, POD, CAT, etc.) of the products are activated, and as free radical purifiers, they can eliminate the free radicals and peroxides of ROS produced under salt stress, avoid the oxidation of these substances on the cell plasma membrane and fatty acid, and thus ensure the integrity of the plasma membrane (Gottschalk, 1994). In this study, when the salt stress concentration is low, the activities of SOD, POD, and CAT increased with the intensification of the salt stress. These three enzymes cooperated with the scavenging of oxygen free radicals and jointly played the role of protecting *X. sorbifolium*. In different plants, SOD activity may be different in response to salt stress. Mahmut et al. observed that the range of SOD activity in almonds is very small compared with that of other antioxidant enzymes after exposure to 20 days of salt stress (Mahmut et al., 2005). Similar results were found in the study of Hossain of two mangrove plants (Hossain et al., 2017). Therefore, we believed that POD and CAT play a critical role in scavenging oxygen free radicals and ROS under salt stress for *X. sorbifolium* species. To protect themselves under salt stress, plants can also synthesize soluble compounds, such as carbohydrates, proline, and betaine, to adjust cellular osmotic conditions and maintain membrane integrity and functions (Lee et al., 2008; Benzarti et al., 2014). Results showed that the proline and soluble protein contents were higher along with the increase of salt concentration, which suggests that the adjustment of osmotic substances played an important role in resisting salt stress. The accumulation of ROS induced by salt also caused increases in antioxidant enzyme activities; in particular, SOD has been suggested to catalyze the conversion of superoxide radicals to molecular oxygen and H_2_O_2_ (Shi et al., 2006). In conclusion, under the condition of salt stress, *X. sorbifolium* can alleviate the damage of reactive oxygen free radicals by increasing the activities of SOD, POD, CAT, and other protective enzymes. There was a certain correlation between the activity of protective enzymes and the lipid peroxidation product MDA. When the stress degree is low, the synergistic effect of protective enzymes in plants keeps the degree of lipid peroxidation at a low level. Once the tolerance limit is exceeded, the activity of protective enzymes will be inhibited, leading to the large production of lipid peroxidation product MDA, which was similar to the findings suggested by Xie et al. (2008).

Another important factor that relates to the salinity response of plants is photosynthesis. The functioning of the photosynthetic apparatus can respond sensitively to environmental disturbances. Therefore, several main photosynthesis-related parameters, such as net photosynthesis rate, Gs, Ci, E, and Wue, have been used to evaluate photosynthesis. Pn usually decreases with rising stress intensity (Koyro, 2006; Wei et al., 2006), whereas in this study, it was found that moderate salt stress could clearly enhance Pn, which may be an adaptive response to salt stress (Yang et al., 2008). Moderate salt could also stimulate the enzyme activity of the scavenging system and promote the photosynthetic capacity. It was also inferred that the high concentration of Na^+^ and Cl^−^ may activate the activity of phosphoenolpyruvate carboxylase and change the pathway of C3 to CAM so that it can adapt to the saline stress better (Beer et al., 1980; Niewiadomska et al., 2004). The reduction of plant Pn under salt stress is generally considered to be the result of a reduction of intracellular CO_2_ concentration caused by stomatal closure, or by non-stomatal factors (Qin et al., 2010). Stomata regulate the uptake of CO_2_ for photosynthesis and the loss of water vapor during transpiration (Belin et al., 2009). Therefore, the decrease in Ci indicates that stomatal limitations are predominant when the NaCl concentration is lower than 140 mM. This kind of disturbance in the stomatal behavior partially resulted from the interruption of water status (Polash et al., 2019). This study proved that Ci truly reduced with Wue. However, the non-stomatal factors depend mainly on the cumulative effects of factors such as leaf water content and osmotic potential, biochemical constituents (Sultana et al., 1999), contents of photosynthetic pigments (Koyro, 2006), and ion toxicity in the cytosol (Ismail et al., 2014). Results showed that under 210 and 280 mM of NaCl, Gs decreased while Ci increased. It indicated that high salt stress might destroy the chloroplast structure in plants and have an influence on the assimilation of CO_2_ and reduction of Pn (Martínez-Ballesta et al., 2000; Apostol et al., 2004; Navarro et al., 2007). This phenomenon further proved that non-stomatal factors become the main factor for the decline in Pn under high salt stress, and there is a strong correlation between the reduction of Pn and POD. It is essential to clarify this coordination between photosynthesis and enzyme activity for evaluating the photosynthetic response to salinity. Consequently, the reduction of Pn is mainly due to the stomatal closure under moderate salt stress, but photosynthesis was seemingly controlled by non-stomatal limitations under the high concentration of NaCl.

According to the correlation analysis and PCA, it can be concluded that there is a strong correlation between enzyme activity, osmotic adjustive substance, and photosynthetic parameters due to salt stress conditions, which consists of a whole response system. Apart from the relation among these different aspects, 140 mM NaCl was considered to be the most suitable condition for the growth of *X. sorbifolium* seedlings. In summary, the data indicated that the growth values of the plant were highest under the CK condition, whereas they can also grow almost normally under the low concentration of NaCl.

Compared with other trees tolerant to salinity, there is some uniqueness of *X. sorbifolium*. First, the growth and root morphology have shown some similar trends under different NaCl concentrations. Leaf wilting and shedding of *X. sorbifolium* seedlings were observed with increasing NaCl salinity, and a decline in plant performance was observed in terms of relative height rate and biomass. Similar results were seen in the study by Bidalia et al. (2016). The root–shoot ratio increased as well with increasing salinity (Meloni et al., 2004). The difference is that the specific leaf area did not change significantly, and more importantly, the root length, root tip, root surface area, and root volume increased under low NaCl stress, indicating the tolerance of roots to salinity (Wu et al., 2010). Our findings in the ion contents are similar to those of a previous study on *Broussonetia papyrifera* under salt stress (Zhang M. et al., 2013). The physiological responses were mostly similar to the study of Zhao et al. (2019), but the change of REC is small and the MDA decreased under high NaCl concentrations. What is more, the change of SOD activity is small as well, and only the POD activity continually increased with increasing salinity. This showed that the enzyme system of *X. sorbifolium* is different from other trees, and the activities of the three enzymes changed in a distinct pattern. The photosynthetic responses are similar to the study of Sun et al. (2011). Compared with high concentrations, low NaCl stress led to the increase in the Pn, Gs, and E, yet decreases the Ci. It can be found that low concentrations of NaCl induced the increase of Pn, Gs, and E but high concentrations of NaCl resulted in the reduction of them. It is a general phenomenon and could indicate the salt tolerance of the plant species. Citrus is not tolerant to salinity, with Pn, Gs, and E continually decreased with increasing salinity (Wu et al., 2010).

## Conclusion

The findings demonstrated in this investigation revealed that *X. sorbifolium* grows better under moderate salt treatment. In brief, even if the concentration was higher than 140 mM, the growth, physiological, and photosynthetic indices of the plant were greatly affected. In particular, 70 mM NaCl promoted the increase of root length and root tips, while Pn reached a maximum value at 140 mM NaCl. Moreover, the plant had the ability of adaptation to the high concentration of NaCl stress, which showed that the proportion of growth in root and aboveground was optimized, antioxidation defense system active was mobilized, as well as membrane lipid peroxidation was effectively inhibited. There is a need to explore for a more concrete correlation among these different response systems to salinity. By the analysis, *X. sorbifolium* performed the best under the 140 mM NaCl treatment. In summary, *X. sorbifolium* Bunge could cope with the salt stress by osmotic, enzyme, and photosynthesis regulations, which could effectively alleviate the damage and provide valuable information that can be used in the popularization of *X. sorbifolium* under a saline environment.

## Data Availability Statement

The original contributions presented in the study are included in the article/Supplementary Material, further inquiries can be directed to the corresponding author.

## Author Contributions

J-WZ and Y-HY conceived the ideas, designed the methodology, collected data, and initiated preparation of the manuscript. Z-LZ, P-LH, N-YC, and K-XX analyzed the data and revised the manuscript. J-WZ led the writing of the manuscript. All authors contributed critically to the drafts and gave final approval for publication.

## Funding

This study was supported by the Science and Technology Project of Henan Province (Project No. 192102110172), the Key Projects of Provincial Universities in Henan Province (Project No. 19A220002), and the Doctoral Research Start-Up Fund project funding (2018HNUAHEDF018 and 2018HNUAHEDF019).

## Conflict of Interest

The authors declare that the research was conducted in the absence of any commercial or financial relationships that could be construed as a potential conflict of interest.

## Publisher's Note

All claims expressed in this article are solely those of the authors and do not necessarily represent those of their affiliated organizations, or those of the publisher, the editors and the reviewers. Any product that may be evaluated in this article, or claim that may be made by its manufacturer, is not guaranteed or endorsed by the publisher.
