# Supplementary material for: Growth, Physiological, and Photosynthetic Responses of Xanthoceras sorbifolium Bunge Seedlings Under Various Degrees of Salinity
Source: Front Plant Sci. 2021 Sep 27;12:730737. doi: 10.3389/fpls.2021.730737 (PMC8504483; doi:10.3389/fpls.2021.730737)
Supplement: Supplementary file 1 [file Data_Sheet_1.doc]

**SUPPLEMENTARY MATERIAL FILE**

**Supplementary Table 1 ∣Principle component analysis for indexes screening**

| **Principal component** | **Eigen value** | **Contribute rate**  **(%)** | **Cumulative Contribute Rate**  **(%)** |
| --- | --- | --- | --- |
| 1 | 5.565 | 46.371 | 46.371 |
| 2 | 2.948 | 24.581 | 70.952 |
| 3 | 1.342 | 11.180 | 82.132 |

**Supplementary Table 2 ∣Component** score coefficient

| **Indexes** | **Component 1** | **Component 2** | **Component 3** |
| --- | --- | --- | --- |
| REC | 0.295 | -0.351 | 0.181 |
| MDA | 0.292 | 0.136 | -0.394 |
| Proline | 0.307 | -0.337 | 0.180 |
| Soluble protein | 0.382 | -0.126 | -0.222 |
| SOD | 0.343 | 0.222 | 0.083 |
| POD | 0.359 | -0.269 | 0.131 |
| CAT | 0.323 | 0.051 | -0.428 |
| Pn | 0.194 | 0.474 | 0.076 |
| Gs | 0.309 | 0.367 | 0.167 |
| Ci | 0.239 | -0.067 | 0.569 |
| E | 0.214 | 0.205 | -0.173 |
| Wue | -0.032 | 0.447 | 0.369 |
